# The association of insulinemic potential of diet and lifestyle with the risk of insulin-related disorders: a prospective cohort study among participants of Tehran Lipid and Glucose Study

**DOI:** 10.1186/s13098-021-00674-z

**Published:** 2021-05-13

**Authors:** Ebrahim Mokhtari, Hossein Farhadnejad, Farshad Teymoori, Parvin Mirmiran, Fereidoun Azizi

**Affiliations:** 1 Nutrition and Endocrine Research Center, Research Institute for Endocrine Sciences, Shahid Beheshti University of Medical Sciences, P.O. Box, 19395-4741 Tehran, Iran; 2 Department of Nutrition, School of Public Health, Iran University of Medical Sciences, Tehran, Iran; 3 Endocrine Research Center, Research Institute for Endocrine Sciences, Shahid Beheshti University of Medical Sciences, Tehran, Iran

**Keywords:** EDIH, ELIH, Insulin-related disorders, Insulin resistance, Insulin insensitivity, Hyperinsulinemia, Β-cells dysfunction

## Abstract

**Background:**

We aim to assess the association of empirical dietary (EDIH) and lifestyle (ELIH) index for hyperinsulinemia with the risk of insulin resistance, hyperinsulinemia, insulin sensitivity, and β-cell dysfunction in Iranian adults.

**Methods:**

In this prospective study, a total of 1244 men and women aged ≥ 20 years were selected among participants of the Tehran lipid and glucose study and followed for 3.2 years. Dietary intakes were assessed using a valid semi-quantitative food frequency questionnaire. Dietary and lifestyle insulinemic potential indices were calculated using dietary intake, body mass index, and physical activity information. Multivariable logistic regression was used to estimate the associated risk of a 3-year incidence of insulin-related disorders.

**Results:**

The mean ± SD age and BMI of all eligible participants (42.7% males) were 43.0 ± 13.0 and 27.4 ± 4.9 in the study's baseline. After adjusting for all potential confounders, participants in the highest tertile of ELIH score had a greater risk of developing hyperinsulinemia (OR:2.42, 95%CI:1.52–3.86, P for trend =  < 0.001), insulin resistance (OR:2.71, 95%CI:1.75–4.18, P for trend =  < 0.001) and insulin insensitivity (OR:2.65, 95%CI: 1.72–4.10, P for trend =  < 0.001) compared with those in the lowest tertile. However, the risk of incident β-cell dysfunction was lower in individuals with a higher score of ELIH in comparison to those with the lowest score (OR:0.30, 95%CI:0.19–0.45, P for trend =  < 0.001).

**Conclusions:**

Empirical lifestyle index for hyperinsulinemia was directly associated with insulin resistance, insulin insensitivity, and hyperinsulinemia and was inversely associated with β-cells dysfunction.

## Background

Insulin-related disorders address three main conditions with complex interrelationships, including Insulin resistance (IR), β-cell dysfunction, and hyperinsulinemia [1–3]. IR, as decreased sensitivity of the peripheral tissue cells to insulin action, [2] and β-cell dysfunction, identified by a dramatic decline in insulin secretion due to pancreatic β-cells hyposensitivity to glucose or disability to overcome the insulin demand because of increased peripheral tissue resistance, [1] are lead to hyperinsulinemia [3]. Persistent hyperinsulinemia and hyperglycemia worsen the IR and β-cell dysfunction by forming a defective cycle during positive feedback. This situation is considered the onset of diabetes mellitus, metabolic syndrome, and cardiovascular risk [4, 5].

The ability of foods to induce postprandial insulin secretion is important for preventing IR and T2DM [6]. Previous studies have suggested a possible link between nutrients [1, 7] and some specific food items [8, 9] and IR and insulin secretion. Because dietary patterns include the interaction between several dietary factors and a better description of food and disease relationships, several studies have been conducted to show the association between dietary patterns and indices and insulin homeostasis [7, 10–12]. Along with the dietary pattern, other lifestyle-related factors, such as obesity and physical activity (PA), are independently related to the progression of insulin-related disorders [13, 14].

Recently, Tabung et al. proposed two dietary and lifestyle insulinemic potential indices to assess the long-term ability of the diet and other lifestyle factors to induce hyperinsulinemia, including the empirical dietary index for hyperinsulinemia (EDIH) and empirical lifestyle index for hyperinsulinemia (ELIH) [15]. It has been shown that the EDIH provides better predictions about both fasting and non-fasting C-peptide concentrations rather than dietary insulin index (DII), indicating that the EDIH may be better in evaluating the dietary impacts of hyperinsulinemia on disease risk. Lee et al., in a recent study, demonstrated that a higher EDIH score is associated with an increased risk of type 2 diabetes mellitus (T2DM) [16] and long-term weight gain [17]. Also, some other studies explored the relationship between EDIH and ELIH with some diseases related to insulin disorders such as colorectal cancer [18], gastrointestinal cancer [19], and multiple myeloma [20, 21]. However, no study has been conducted to investigate the relationship between indices of the insulinemic potential of diet and lifestyle with insulin-related disorders.

In this study, we aim to assess the association of EDIH and ELIH with the risk of IR, hyperinsulinemia, insulin sensitivity, and β-cell dysfunction in Iranian adults.

## Materials and methods

### Study participants

**Fig. 1:**
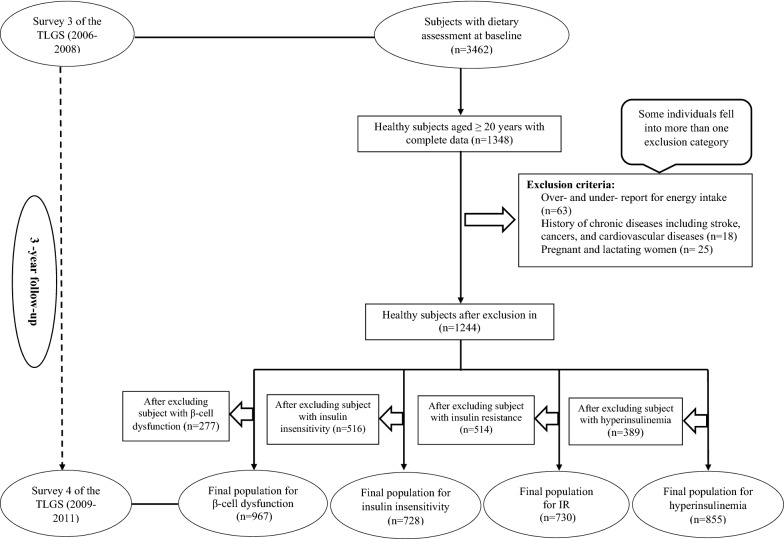
The diagram of the study participants in the Tehran Lipid and Glucose Study

The current study was conducted within the Tehran lipid, and glucose study (TLGS), which started in 1999 in Tehran city, and its data are collected prospectively at 3-year intervals [22]. The study inclusion criteria included age ≥ 20 years, no insulin-related disorders as the baseline of study (third examination), having complete data on dietary intakes, anthropometric and biochemistry variables, no history of myocardial infarction (MI), stroke and cancer, no pregnancy, and lactation. Also, the exclusion criteria of the current study were over-reporting and underreporting on dietary intakes, pregnancy or lactation during follow-up, and missing during follow-up time because of lack of cooperation or other reasons. In the third survey of the TLGS (2006–08), of 12 523 participants, 3462 were randomly selected for dietary assessment. For the present study, 1348 men and women aged ≥ 20 years were selected with complete insulin data. Individuals with a history of MI or stroke or cancer (n = 18), those who reported daily energy intakes outside the range of 800–4200 kcal/day (n = 63), and pregnant and lactating women (n = 25) were excluded; some individuals fell into more than one exclusion category. Finally, 1244 participants were followed until Survey IV (2009–11), with a median follow-up period of 3.2 years (interquartile range [IQR] 2.0–3.0 years). Data were analyzed for assessing the association between EDIH and ELIH and incidence of insulin-related disorders, including hyperinsulinemia (n = 855), IR (n = 730), β-cell dysfunction (n = 967), and insulin insensitivity (n = 728) after excluding the participants who had these insulin-related disorders at baseline of the current study (Fig. 1).

### Sample size calculation

The sample size for the present study was calculated using the G power software 3.1.9.4 version. The minimum sample size for EDIH was 507 participants computed using the 80% power, 5% level of significance, OR = 1.87 for the highest vs. lowest categories of EDIH for each insulin disorders (this OR previously observed for diabetes in participants who were in the highest vs. lowest quartiles of EDIH [23]), and the incident rate of hyperinsulinemia as nearly 20% that previously reported in TLGS study [24]. We used all eligible participants of the TLGS study, which was more than 507 participants for every insulin disorder as an outcome.

### Physical activity assessment

The modified and validated version of the Modified Activity Questionnaire (MAQ) for the Iranian population was used to assess participants' PA status. Validity was assessed via comparing data between the means of 2 MAQs and the means of four physical activity records with the Spearman correlation coefficients [25]. This questionnaire consists of questions in two categories, including leisure activities and work-related activities. Individuals were asked to report the frequency and time spent for these two categories during the past 12 months as light, moderate, hard, and very hard intensity. To calculate the level of activities with different intensities, each activity was weighed in terms of MET (metabolic equivalent) based on their relative intensity. The number of times and duration of each activity are recorded in the questionnaire. Physical activity level was calculated as the amount of MET multiplied by the duration of activity in minutes multiplied by the number of activities per week. Finally, levels of PA are expressed as metabolic equivalent hours per week.

### Demographic, anthropometric, and lifestyle measures

Demographic information was assessed by skilled interviewers using a pretested questionnaire. In this questionnaire, participants were asked to collect data on several variables including age, sex, smoking status as a current smoker (daily or occasionally or ex-smokers) or non-smokers, educational level (graduated/non- graduated), medical history (diabetes, hypertension, myocardial infarction, stroke, and cancers), medication use (anti-diabetic and glucose-lowering drugs). Weight was measured with light clothing and accuracy of up to 100 g using a SECA digital weighing scale (Seca 707; Seca Corporation, Hanover, Maryland; range, 0.1–150 kg). Height was measured in a standing position, without shoes and shoulders in normal alignment, using a stadiometer with a minimum of 1 mm. Body mass index (BMI) was calculated as weight (Kg)/height^2^ (m^2^).

### Biochemical measurements

Blood samples of all subjects were collected after 12–14 h of overnight fasting in a steady-state sitting position between 7:00 and 9.00 AM., immediately centrifuged within 30–45 min of collection. All samples were analyzed at the TLGS research laboratory on collection using Selectra 2 auto-analyzer (Vital Scientific, Spankeren, Netherlands). Fasting blood sugar (FBS) was measured using an enzymatic colorimetric method with glucose oxidase. Inter/intra-assay coefficient variations for FBS were both 2.2% for FBS. Fasting Insulin was measured via electrochemiluminescence immunoassay (ECLIA), using Roche Diagnostics kits and Roche/Hitachi Cobas e-411 analyzer (Gmbh, manhim, Germany). Inter- and intra-assay coefficient variations for insulin were 1.2 and 3.5, respectively.

### Dietary intake assessment

Dietary intakes were assessed using a valid and reliable 168-item semi-quantitative food frequency questionnaire (FFQ). The FFQ validity was previously fulfilled by comparing food groups values derived from the questionnaire with values estimated by twelve 24-h dietary recall surveys [26, 27]. This FFQ was a Willett-format questionnaire contains 168 food items. During a face-to-face interview, the frequency of consumption for each food item during the past year on a daily, weekly, or monthly basis was collected by trained and skilled dieticians. According to the most frequently consumed items in Iran's national food consumption survey, the food items were chosen. Portion sizes of consumed foods reported in domestic measures were then transformed to gram scale. The United States Department of Agriculture (USDA) food composition table (FCT) is used to compute energy and nutrients content. The Iranian FCT was used for some local food items which were not available in USDA FCT. Dietary intakes in the third phase (2008–2011) of TLGS were considered as exposure at baseline.

### Calculation of indices

Dietary data derived from FFQ were used to calculate insulinemic scores. Since consumption of alcoholic drinks such as wine and liquor is unusual in the Iranian population due to religious considerations and was not reported in the TLGS study, we do not include them in calculating indices. Calculating the EDIH and ELIH has been explained elsewhere [2]. As we have no food items as low energy beverages and cream soup in our FFQ, we exclude them in the calculation.

So we calculated EDIH score with 15 instead of 18 food parameters in two groups according to their potential to induce or suppress hyperinsulinemia, including processed meat (sausage), red meat (beef, or lamb), fish (canned tuna, or fish), margarine, poultry (chicken or turkey with or without skin), French fries, high-energy beverages (cola with sugar, carbonated beverages with sugar, fruit punch drinks), tomatoes, low-fat dairy products (skimmed or low-fat milk and yogurt) and eggs (positive association). Furthermore, coffee, green leafy vegetables (cabbage, spinach, or lettuce), whole fruits, and high-fat dairy products (whole milk, cream, cream cheese, and other cheese) (inverse association).

In the same way, the ELIH score was calculated with 11 instead of 14 dietary and lifestyle factors, including BMI, margarine, butter, red meat, and fruit juice (apple juice, cantaloupe juice, orange juice, or other fruit juice) with a positive association and coffee, whole fruit, PA, high-fat dairy products, snacks and salad dressing with the inverse association. The food groups' daily intakes (serving size) and lifestyle factors values multiplied by specific proposed regression coefficients for weighting. Finally, to calculate total scores, all weighted food group intakes lifestyle factors were summed and then divided by 1000 to decline the scores' magnitude, which eases the results' interpretation.

### Outcome ascertainment

#### Insulin resistance (IR)

Homeostatic Model Assessment of Insulin Resistance (HOMA-IR) was used to assess IR (HOMA-IR = FBS (mmol/L) × Insulin (μU/mL)/22.5). HOMA-IR ≥ 2.17 and 1.85 were determined as criteria for IR for men and women, respectively.

#### Hyperinsulinemia

Fasting Insulin concentrations ≥ 9.16 and 11.13 are considered as criteria for hyperinsulinemia for men and women, respectively.

#### Insulin insensitivity

Homeostatic Model Assessment of Insulin sensitivity (HOMA-S) was used to assess Insulin insensitivity (HOMA-S = (1/HOMA-IR) × 100), where HOMA-S ≤ 46.1 and 54.1 are considered as criteria for insulin insensitivity for men and women, respectively.

#### β-cell dysfunction

It was determined using the Homeostatic Model Assessment of β-cell function (HOMA-β) as follows:

(HOMA-β = Insulin (μU/mL) × 20/FBS (mmol/L)—3.5). HOMA-β ≤ 67.1 and 86.2 are defined as criteria for β-cell dysfunction for men and women, respectively.

### Statistical analysis

Data were analyzed using the Statistical Package for Social Sciences (version 20.0; SPSS Inc, Chicago, IL). Histogram charts and Kolmogorov–Smirnov analysis were used to assess the normality of variables. Participants were categorized according to EDIH and ELIH tertiles cutoff points. Baseline characteristics of individuals were expressed for continuous and categorical variables as mean ± SD or median (25–75) interfertile range (IQR) and percentage, respectively. Trends of qualitative and quantitative variables across tertiles of ELIH and EDIH ratio (as the median value in each tertile) were tested using Chi-square and linear regression. Multivariable logistic regression was used to estimate the risk of 3-year incidence of insulin-related disorders, with IR, hyperinsulinemia, insulin insensitivity, and β-cell dysfunction as dependent variables and the EDIH and ELIH scores as independent variables; odds ratio (OR) and 95% confidence interval (CI) were reported. All of the regression models were adjusted for age, sex, smoking status, PA, BMI, energy intake, education level, hypertension, and diabetes. P-values < 0.05 were considered as statistically significant. Furthermore, in an additional step, sensitivity analysis was performed with excluding individuals with a history of diabetes at baseline.

## Results

The mean ± SD age and BMI of all eligible participants (42.7% males) were 43.0 ± 13.0 and 27.4 ± 4.9, respectively. The median (IQR) EDIH and ELIH of participants were 0.14 (0.05–0.28) and 1.33 (1.16–1.55). After excluding each of the insulin disorders at baseline, during 3.2 years of follow-up, the incidence of hyperinsulinemia, β-cell dysfunction, insulin insensitivity, and IR was 20.0, 24.5, 29.9, and 30%, respectively.

The baseline characteristics and dietary intakes of participants according to tertiles of the ELIH are shown in Table 1. The BMI, T2DM, FBS, fasting serum insulin, HOMA-IR, HOMA-β, hyperinsulinemia, and insulin insensitivity increased significantly across ELIH score tertiles. In contrast, male percent, PA level, smoking, and percent of graduated participants, HOMA-S, and β-cell dysfunction decreased (p for trend =  < 0.05). Also, dietary intake of energy, fat, red and processed meat, and scores of ELIH and EDIH increased across these tertiles; however, dietary intake of carbohydrate, high-fat dairy, and fruits decreased across tertiles of ELIH score (p for trend < 0.05).

**Table 1:** Baseline characteristics of 1244 participants of study population across tertiles (T) of the empirical lifestyle index for hyperinsulinemia

	Empirical lifestyle index for hyperinsulinemia			
	T1 (n = 412)	T2 (n = 411)	T3 (n = 411)	P for trend
Age ± years	42.3 ± 14.1	43.1 ± 12.6	43.7 ± 12.0	0.132
Male (%)	195 (47.3)	183 (44.5)	152 (37.0)	0.008
Body mass index (kg/m2)	23.5 ± 2.7	26.9 ± 2.7	31.8 ± 4.8	< 0.001
Physical activity (MET-h/week)	33.0 (14.0–77.3)	21.4 (9.7–47.6)	23.6 (10.0–43.7)	< 0.001
Current smokers (%)	63 (15.3)	51 (12.4)	37 (9.0)	0.021
Education Level (graduated), (%)	129 (31.3)	108 (26.3)	78 (19.0)	< 0.001
Hypertension, n (%)	46 (11.2)	58 (14.1)	67 (16.3)	0.079
Diabetes, n (%)	23 (5.6)	30 (7.3)	41 (10.0)	0.051
Biochemical data				
Fasting serum insulin(mU/mL)	7.27 ± 4.32	8.59 ± 4.00	11.72 ± 7.24	< 0.001
Fasting blood sugar (mmol/l)	4.97 ± 1.33	5.1 ± 1.33	5.33 ± 1.53	< 0.001
HOMA-IR	1.35 (0.95–1.93)	1.76 (1.21–2.40)	2.29 (1.59–3.51)	< 0.001
HOMA-B	105.7 (73.9–149.2)	120.0 (81.8–167.1)	146.0 (90.6–196.8)	< 0.001
HOMA-S	83.6 ± 46.7	66.8 ± 42.7	49.9 ± 29.5	< 0.001
Hyperinsulinemia, n (%)	69 (16.7)	113 (27.5)	204 (49.6)	< 0.001
Insulin resistance, n (%)	104 (25.2)	157 (38.2)	247 (60.1)	< 0.001
Insulin insensitivity, n (%)	104 (25.2)	158 (38.4)	248 (60.3)	< 0.001
β-cell dysfunction, n (%)	109 (26.5)	88 (21.4)	77 (18.7)	0.028
Nutrient Intake				
Energy(Kcal/d)	2202 ± 689	2224 ± 688	2302 ± 757	0.041
Carbohydrate(% of energy)	59.0 ± 7.0	58.4 ± 6.8	56.2 ± 7.7	< 0.001
Protein(% of energy)	13.5 ± 2.1	13.7 ± 2.5	13.7 ± 2.5	0.348
Fat(% of energy)	30.2 ± 6.6	30.4 ± 6.7	32.6 ± 7.7	< 0.001
Food groups				
Low-fat dairy (serving/d)	1.08 ± 0.90	0.97 ± 0.81	1.01 ± 0.80	0.297
High-fat dairy (serving/d)	1.34 ± 1.04	1.09 ± 0.69	1.06 ± 0.73	< 0.001
Refined grain(serving/d)	5.17 ± 3.73	5.25 ± 3.96	5.34 ± 3.56	0.524
Red and processed meat(serving/d)	0.59 ± 0.43	0.80 ± 0.61	1.08 ± 1.00	< 0.001
Fruits(serving/d)	3.51 ± 2.75	3.33 ± 2.79	3.13 ± 2.37	0.041
Vegetables(serving/d)	2.88 ± 1.97	2.94 ± 2.07	3.07 ± 2.18	0.197
Insulin scores				
EDIH	0.09 (0.02–0.19)	0.15 (0.06–0.27)	0.20 (0.08–0.44)	< 0.001
ELIH	1.06 ± 0.12	1.34 ± 0.06	1.70 ± 0.20	< 0.001

Data are presented as mean ± SD for continuous variable and number (percent) for categorical variables

*HOMA-IR* Homeostatic Model Assessment for Insulin Resistance, *HOMA-B* Homeostatic Model Assessment for β-cell function, *HOMA-S* Homeostatic Model Assessment for insulin sensitivity, *EDIH* Empirical dietary index for hyperinsulinemia, *ELIH* Empirical lifestyle index for hyperinsulinemia

Baseline characteristics and dietary intakes across tertiles of the EDIH among all eligible participants in the baseline of the study are presented in Table 2. By increasing the score of EDIH, the male percent, smoking, and dietary intakes of energy, fat, low-fat dairy, refined grain, red and processed meat, vegetables, and scores of ELIH and EDIH increased (p for trend =  < 0.05), whereas, age, BMI, percent of hypertension and diabetes, FBS, and dietary intake of carbohydrate and high-fat dairy decreased (p for trend =  < 0.05).

**Table 2:** Baseline characteristics of 1244 participants of study population across tertiles (T) of the empirical dietary index for hyperinsulinemia

	Empirical dietary index for hyperinsulinemia			
	T1(n = 415)	T2(n = 415)	T3(n = 414)	P for trend
Age ± years	45.7 ± 13.5	42.2 ± 12.5	40.9 ± 12.4	< 0.001
Male (%)	155 (37.3)	177 (42.7)	199 (48.1)	0.008
Body mass index (kg/m2)	27.8 ± 4.9	27.6 ± 5.1	26.8 ± 4.7	0.004
Physical activity (MET-h/week)	27.7 (11.7–55.5)	25.3 (9.6–55.5)	24.3 (11.3–49.0)	0.754
Current smokers (%)	35 (8.4)	53 (12.8)	63 (15.2)	0.009
Education Level (graduated), (%)	92 (22.2)	112 (27.0)	116 (28.0)	0.163
Hypertension, n (%)	71 (17.1)	60 (14.5)	41 (9.9)	0.010
Diabetes, n (%)	44 (10.6)	31 (7.5)	20 (4.8)	0.008
Biochemical data				
Fasting serum insulin(mU/mL)	9.29 ± 5.40	9.19 ± 6.38	9.06 ± 5.22	0.570
Fasting blood sugar (mmol/l)	5.23 ± 1.57	5.15 ± 1.45	5.00 ± 1.14	0.017
HOMA-IR	1.80 (1.25–2.69)	1.70 (1.19–2.58)	1.69 (1.15–2.50)	0.190
HOMA-B	117.2 (79.2–167.1)	118.8 (81.9–165.6)	127.0 (81.9- 177.8)	0.687
HOMA-S	65.2 ± 42.2	67.2 ± 44.6	68.0 ± 40.6	0.391
Hyperinsulinemia, n (%)	123 (29.6)	130 (31.3)	136 (32.9)	0.608
Insulin resistance, n (%)	186 (44.8)	165 (39.8)	162 (39.1)	0.197
Insulin insensitivity, n (%)	187 (45.1)	166 (40.0)	162 (39.1)	0.181
β-cell dysfunction, n (%)	100 (24.1)	88 (21.2)	88 (21.3)	0.527
Nutrient intake				
Energy(Kcal/d)	1990 ± 632	2162 ± 701	2566 ± 682	< 0.001
Carbohydrate(% of energy)	59.9 ± 7.2	58.4 ± 6.8	55.2 ± 7.0	< 0.001
Protein(% of energy)	13.4 ± 2.1	13.9 ± 2.3	13.7 ± 2.6	0.056
Fat(% of energy)	29.4 ± 7.0	30.4 ± 6.7	33.4 ± 6.9	< 0.001
Food groups				
Low-fat dairy (serving/d)	0.86 ± 0.73	1.01 ± 0.79	1.18 ± 0.94	< 0.001
High-fat dairy (serving/d)	1.33 ± 1.00	1.01 ± 0.67	1.15 ± 0.80	0.030
Refined grain(serving/d)	4.86 ± 3.77	5.06 ± 3.71	5.81 ± 3.71	< 0.001
Red and processed meat(serving/d)	0.54 ± 0.41	0.80 ± 0.61	1.13 ± 0.98	< 0.001
Fruits(serving/d)	3.48 ± 2.92	3.24 ± 2.32	3.25 ± 2.65	0.288
Vegetables(serving/d)	2.44 ± 1.51	3.02 ± 1.94	3.39 ± 2.54	< 0.001
Insulin scores				
EDIH	0.0 ± 0.06	0.14 ± 0.03	0.45 ± 0.23	< 0.001
ELIH	1.27 ± 0.27	1.34 ± 0.27	1.48 ± 0.30	< 0.001

Data are presented as mean ± SD for continuous variable and number (percent) for categorical variables

*HOMA-IR* Homeostatic Model Assessment for Insulin Resistance, *HOMA-B* Homeostatic Model Assessment for β-cell function, *HOMA-S* Homeostatic Model Assessment for insulin sensitivity, *EDIH* Empirical dietary index for hyperinsulinemia, *ELIH* Empirical lifestyle index for hyperinsulinemia

The association between ELIH and EDIH scores with the risk of insulin-related disorders, including hyperinsulinemia, IR, β-cell dysfunction, and insulin insensitivity, is indicated in Table 3. ELIH score showed a significant association with the risk of each insulin-related disorder in all adjusted models. In the final model, after adjusting for all potential confounders including age, sex, smoking status, PA, BMI, energy intake, education level, hypertension, and diabetes, participants in the highest tertile of ELIH score had a greater risk of developing hyperinsulinemia (OR:2.42, 95%CI:1.52–3.86, P for trend =  < 0.001), IR (OR:2.71, 95%CI:1.75–4.18, P for trend =  < 0.001) and insulin insensitivity (OR:2.65, 95%CI: 1.72–4.10, P for trend =  < 0.001) compared with those in the lowest tertile. However, the risk of incident β-cell dysfunction was lower in individuals with a higher score of ELIH in comparison to those with the lowest score (OR:0.30, 95%CI:0.19–0.45, P for trend =  < 0.001). We also have assessed the association of EDIH scores with the risk of insulin-related disorders. Based on the results of all three models (Table 3), there was no significant association between EDIH score and risk of hyperinsulinemia, IR, β-cell dysfunction, and insulin insensitivity.

**Table 3:** The association between the insulin response dietary and lifestyle indices and incidence of insulin related disorders

	Tertiles of ELIH			P_trend	Tertiles of EDIH			P_trend
	T1	T2	T3		T1	T2	T3	
Hyperinsulinemia								
Median score	1.04	1.28	1.55		0.01	0.14	0.37	
Case/Total	36/283	63/283	71/282		68/285	45/285	58/285	
Model 1^a^	1.00 (Ref.)	2.05 (1.29–3.26)	2.46 (1.55–3.90)	< 0.001	1.00 (Ref.)	0.60 (0.38–0.93)	0.74 (0.48–1.14)	0.609
Model 2^b^	1.00 (Ref.)	2.06 (1.30–3.28)	2.41 (1.51–3.85)	< 0.001	1.00 (Ref.)	0.61 (0.38–0.95)	0.75 (0.47–1.19)	0.633
Model 3^c^	1.00 (Ref.)	2.07 (1.30–3.29)	2.42 (1.52–3.86)	< 0.001	1.00 (Ref.)	0.60 (0.38–0.95)	0.75 (0.47–1.19)	0.618
Insulin resistance								
Median score	1.03	1.27	1.51		0.02	0.15	0.40	
Case/Total	43/242	81/242	93/241		74/244	67/243	76/243	
Model 1^a^	1.00 (Ref.)	2.28 (1.48–3.51)	2.80 (1.82–4.29)	< 0.001	1.00 (Ref.)	0.98 (0.66–1.48)	1.16 (0.78–1.74)	0.434
Model 2^b^	1.00 (Ref.)	2.28 (1.48–3.51)	2.74 (1.78–4.21)	< 0.001	1.00 (Ref.)	0.94 (0.62–1.43)	1.09 (0.70–1.69)	0.495
Model 3^c^	1.00 (Ref.)	2.30 (1.49–3.55)	2.71 (1.75–4.18)	< 0.001	1.00 (Ref.)	0.94 (0.62–1.44)	1.09 (0.70–1.70)	0.475
β-cell dysfunction								
Median score	1.09	1.35	1.66		0.02	0.15	0.39	
Case/Total	110/319	72/320	53/320		100/323	67/322	71/322	
Model 1^a^	1.00 (Ref.)	0.51 (0.35–0.74)	0.31 (0.20–0.46)	< 0.001	1.00 (Ref.)	0.57 (0.39–0.83)	0.70 (0.48–1.01)	0.229
Model 2^b^	1.00 (Ref.)	0.51 (0.35–0.75)	0.32 (0.21–0.48)	< 0.001	1.00 (Ref.)	0.60 (0.41–0.88)	0.76 (0.51–1.15)	0.430
Model 3^c^	1.00 (Ref.)	0.52 (0.36–0.76)	0.30 (0.19–0.45)	< 0.001	1.00 (Ref.)	0.60 (0.40–0.89)	0.79 (0.52–1.20)	0.437
Insulin insensitivity								
Median score	1.03	1.27	1.51		0.02	0.15	0.40	
Case/Total	43/240	80/241	93/242		74/243	67/243	75/242	
Model 1^a^	1.00 (Ref.)	2.26 (1.47–3.47)	2.75 (1.79–4.21)	< 0.001	1.00 (Ref.)	0.96 (0.64–1.45)	1.12 (0.75–1.68)	0.497
Model 2^b^	1.00 (Ref.)	2.26 (1.47–3.49)	2.69 (1.75–4.13)	< 0.001	1.00 (Ref.)	0.92 (0.60–1.39)	1.03 (0.66–1.59)	0.803
Model 3^c^	1.00 (Ref.)	2.28 (1.48–3.53)	2.65 (1.72–4.10)	< 0.001	1.00 (Ref.)	0.92 (0.60–1.40)	1.03 (0.66–1.61)	0.796

^a^Model 1: adjusted for age and sex

^b^Model 2: adjusted for model 1 and energy intake, smoking, education level (for both), body mass index and physical activity (only for EDIH)

^c^Model 3: adjusted for model 2 and baseline diabetes and hypertension

Also, in sensitivity analysis, the findings of the previous analysis were repeated while the odds ratios were strengthened (Table 4).

**Table 4:** The association between the insulin response dietary and lifestyle indices and incidence of insulin related disorders after excluding the diabetic patients in the baseline of study

	Tertiles of ELIH			P_trend	Tertiles of EDIH			P_trend
	T1	T2	T3		T1	T2	T3	
Hyperinsulinemia								
Median score	1.04	1.28	1.55		0.02	0.14	0.37	
Case/Total	34/273	59/264	68/258		64/261	41/268	57/272	
Model 1^a^	1.00 (Ref.)	2.14 (1.33–3.45)	2.66 (1.66–4.28)	< 0.001	1.00 (Ref.)	0.55 (0.35–0.88)	0.72 (0.47–1.12)	0.331
Model 2^b^	1.00 (Ref.)	2.15 (1.33–3.46)	2.62 (1.62–4.22)	< 0.001	1.00 (Ref.)	0.56 (0.35–0.89)	0.73 (0.45–1.17)	0.376
Model 3^c^	1.00 (Ref.)	2.15 (1.33–3.46)	2.61 (1.62–4.22)	< 0.001	1.00 (Ref.)	0.56 (0.35–0.89)	0.73 (0.45–1.16)	0.542
Insulin resistance								
Median score	1.03	1.27	1.51		0.02	0.15	0.40	
Case/Total	40/236	74/234	89/230		66/232	64/237	73/236	
Model 1^a^	1.00 (Ref.)	2.26 (1.45–3.52)	2.95 (1.90–4.58)	< 0.001	1.00 (Ref.)	1.03 (0.68–1.57)	1.22 (0.80–1.84)	0.314
Model 2^b^	1.00 (Ref.)	2.25 (1.44–3.51)	2.87 (1.84–4.47)	< 0.001	1.00 (Ref.)	0.97 (0.63–1.50)	1.10 (0.70–1.74)	0.597
Model 3^c^	1.00 (Ref.)	2.24 (1.43–3.50)	2.83 (1.82–4.26)	< 0.001	1.00 (Ref.)	0.96 (0.62–1.48)	1.10 (0.70–1.73)	0.614
β-cell dysfunction								
Median score	1.09	1.35	1.66		0.02	0.15	0.39	
Case/Total	107/314	68/314	43/304		89/309	64/314	68/317	
Model 1^a^	1.00 (Ref.)	0.51 (0.35–0.73)	0.29 (0.19–0.44)	< 0.001	1.00 (Ref.)	0.60 (0.41–0.88)	0.76 (0.52–1.11)	0.566
Model 2^b^	1.00 (Ref.)	0.51 (0.35–0.74)	0.30 (0.19–0.45)	< 0.001	1.00 (Ref.)	0.64 (0.43–0.96)	0.84 (0.55–1.27)	0.592
Model 3^c^	1.00 (Ref.)	0.52 (0.36–0.76)	0.30 (0.19–0.45)	< 0.001	1.00 (Ref.)	0.66 (0.44–0.98)	0.84 (0.55–1.27)	0.581
Insulin insensitivity								
Median score	1.03	1.27	1.51		0.02	0.15	0.40	
Case/Total	40/234	73/233	89/231		66/231	64/237	72/235	
Model 1^a^	1.00 (Ref.)	2.23 (1.43–3.48)	2.89 (1.86–4.50)	< 0.001	1.00 (Ref.)	1.01 (0.67–1.54)	1.17 (0.77–1.78)	0.403
Model 2^b^	1.00 (Ref.)	2.23 (1.43–3.49)	2.82 (1.81–4.39)	< 0.001	1.00 (Ref.)	0.95 (0.61–1.46)	1.04 (0.66–1.63)	0.793
Model 3^c^	1.00 (Ref.)	2.31 (1.42–3.48)	2.78 (1.78–4.34)	< 0.001	1.00 (Ref.)	0.93 (0.60–1.44)	1.03 (0.65–1.62)	0.812

^a^Model 1: adjusted for age and sex

^b^Model 2: adjusted for model 1 and energy intake, smoking, education level (for both), body mass index and physical activity (only for EDIH)

^c^Model 3: adjusted for model 2 and baseline hypertension

## Discussion

We investigated the association between EDIH and ELIH and insulin-related disorders incidence in the present population-based cohort study. Our findings showed a 142, 171, and 165% higher risk of hyperinsulinemia, IR, insulin insensitivity, and 70% lower risk of β-cell dysfunction among participants in the highest tertile of the ELIH score compared with those in the lowest tertile. However, in our study, the EDIH score was not associated with the risk of the above-mentioned insulin-related disorders.

Nowadays, the assessing of the relationship between diet alone or combined with other lifestyle factors and risk of various chronic diseases is becoming one of the most accepted aspects of nutritional studies because it is revealed that this complex involves the interrelationships between different key factor and so provide a comprehensive insight into this regard [11, 23, 28–31]. Recently, EDIH and ELIH proposed to predict the body's insulin response to dietary and lifestyle factors. Since hyperinsulinemia has been identified as an early metabolic dysfunction indicator previously [3], several studies explored the association of these two indices with the risk of chronic diseases, in which impaired insulin balance plays an important role in their pathogenesis [16–21]. In this regard, a significant association has been observed between these indices and the risk of some insulin-related malignancies, including colorectal cancer [18], digestive system cancer [19], and multiple myeloma [20, 21], previously. Two prospective studies with long-term follow-up have also demonstrated that a higher EDIH score is associated with an increased risk of diabetes [19] and substantial weight gain [20]. However, in our study, the findings on the association of EDIH and the risk of insulin resistance disorders was not significant, this may be due to the fact that the onset of chronic disease symptoms may take a long time, and despite its salient features in the development of hyperinsulinemia, this dietary pattern alone cannot predict the risk of insulin-related disorders in the short-term period.

We have observed a strong positive association between a higher ELIH score and hyperinsulinemia, IR, and insulin insensitivity. ELIH is an index that combined two important lifestyle factors, BMI and PA, with diet. According to the Tabung et al. study, People on a diet with higher hyperinsulinemic potential are at greater risk of substantial long-term weight gain and higher BMI than others [17]. Besides, based on previous studies, overweight and obesity can increase the risk of IR, compensatory insulin hypersecretion, and the destruction of pancreatic beta cells. [32–34]. PA, either directly, by increasing the production and secretion of anti-inflammatory cytokines that reduce systemic inflammation and increase insulin sensitivity in muscles [34, 35], or indirectly by helping to lose weight, and also keeping an appropriate balance between abdominal and intramuscular adipose tissue [36], can regulate the insulin response of the body. Therefore, similar to the diet, each BMI and PA alone is a stronger predictor of insulin-related disorders, and as expected that the ELIH score, which addresses the collective contributions of the three factors, though can better predicts the risk of insulin-related disorders rather than the dietary index (EDIH) in the relatively short-term period follow-up of our study.

Despite the other three disorders, our findings showed a lower risk of β-cells dysfunction among the participants in the highest vs. those in the lowest tertiles of ELIH scores. According to the definition of HOMA-β, the higher ratio of insulin to FBS indicates a better function of the β-cells, which is what happened in the present study. In our study, despite slight changes in FBS with increasing the ELIH score, increasing insulin concentration reduced beta-cell dysfunction incidence. This suggests that a hyperinsulinemic lifestyle may delay beta cell destruction for a short or midterm. Previous studies have shown that hyperinsulinemia can increase insulin secretion by increasing intracellular signaling and sensitivity of beta cells in response to insulinotropic agents and beta-cell hypertrophy [37, 38]. In any case, these compensatory processes are transient, while following a hyperinsulinemic lifestyle, in the long run, increases insulin resistance and beta-cell depletion, leading to progressive destruction and the development of diabetes [39].

This study had several strengths. This is the first study that assessed the association of EDIH and ELIH with odds of insulin-related disorders to the best of our knowledge. The current study's major other strengths were the prospective setting, the use of valid and reliable food frequency, and PA questionnaires for dietary and PA assessments. On the other side, our study was not without limitations. In the present study, Like all nutritional studies using FFQ for dietary assessment, some measurement errors are inevitable. The golden standard method to assess the β-cells function is the hyperglycemic clamp technique, but due to our limitations, the best method available for us was the homeostatic model assessment. Even though this method seems suitable for epidemiologic studies, we tried to cover this limitation through accurate and standard fasting insulin and glucose assessment. Also, the length of follow-up in this study may not be sufficient to evaluate some insulin-related outcomes, particularly beta-cell dysfunction. It means that if the follow-up period lasting a longer time, the findings may be different. For example, most probably, β-cells dysfunction would be increased instead of decrease. Finally, there is possible residual confounding that we cannot exclude due to unknown or unmeasured factors.

## Conclusion

Our finding suggested that a higher ELIH score is associated with increased odds of IR, insulin insensitivity, hyperinsulinemia, and a decreased risk of β-cells dysfunction. Further epidemiological studies are needed to address the role of the insulinemic potential of diet and lifestyle in the odds of insulin-related disorders and their potential mechanisms.

## Data Availability

The data underlying this article will be shared at reasonable request to the corresponding author.

## Abbreviations

EDIH: Empirical dietary index for hyperinsulinemia
ELIH: Empirical lifestyle index for hyperinsulinemia
FBS: Fasting blood sugar
IR: Insulin resistance
T2DM: Type 2 diabetes mellitus
HbA1c: Hemoglobin A1c
HOMA-IR: Homeostatic model assessment for insulin resistance
HOMA-S: Homeostatic model assessment for insulin sensitivity
HOMA-β: Homeostatic model assessment for β-cells function
DII: Dietary insulin index
DIL: Dietary insulin load
DASH: Dietary Approaches to Stop Hypertension
PAI-1: Plasminogen activator inhibitor-1
TNF-α: Tumor Necrosis Factor-α
ECLIA: Electrochemiluminescence immunoassay
USDA: United States Department of Agriculture
FCT: Food composition table
BMI: Body mass index
PA: Physical activity
FFQ: Food Frequency Questionnaire
MAQ: Modifiable Activity Questionnaire
CI: Confidence interval
SD: Standard deviation
IQR: Interquartile range
TLGS: Tehran lipid and glucose study
